# Chronic myeloid leukemia stem cells

**DOI:** 10.1038/s41375-019-0490-0

**Published:** 2019-05-24

**Authors:** Mohammad Houshmand, Giorgia Simonetti, Paola Circosta, Valentina Gaidano, Alessandro Cignetti, Giovanni Martinelli, Giuseppe Saglio, Robert Peter Gale

**Affiliations:** 1 Department of Clinical and Biological Sciences, University of Turin, Turin, Italy; 2 Istituto Scientifico Romagnolo per lo Studio e la Cura dei Tumori (IRST) IRCCS, Meldola, FC Italy; 3 Ospedale Mauriziano di Torino, Turin, Italy; 4 Imperial College London, London, UK

**Keywords:** Cancer stem cells, Cell signalling

## Abstract

Chronic myeloid leukemia (CML) is caused by *BCRABL1* in a cell with the biological potential, intrinsic or acquired, to cause leukemia. This cell is commonly termed the CML leukemia stem cell (LSC). In humans a CML LSC is operationally-defined by ≥1 in vitro or in vivo assays of human leukemia cells transferred to immune-deficient mice. Results of these assays are sometimes discordant. There is also the unproved assumption that biological features of a CML LSC are stable. These considerations make accurate and precise identification of a CML LSC difficult or impossible. In this review, we consider biological features of CML LSCs defined by these assays. We also consider whether CML LSCs are susceptible to targeting by tyrosine kinase inhibitors (TKIs) and other drugs, and whether elimination of CML LSCs is needed to achieve therapy-free remission or cure CML.

## Introduction

Chronic myeloid leukemia (CML) is a blood cancer caused by *BCRABL1* in a cell with the biological ability, intrinsic or acquired, to cause leukemia [1]. *BCRABL1* encodes a 210 KD chimeric protein (P210^*BCRABL1*^) with constitutive tyrosine-kinase activity [2, 3]. By various incompletely defined mechanisms, this abnormality results in expansion of the leukemia clone [4]. In *chronic-phase* CML, proliferation is regulated, such that the leukemia cells mature normally and respond appropriately to normal regulators, such as granulocyte-colony-stimulating and macrophage-colony-stimulating factors (G-CSF and G/M-CSF) and to infection [5]. There are simply too many of them. Also, in rare persons with cyclic neutropenia and CML levels of blood leukemia granulocytes also cycle indicating that they respond to normal regulation of granulocyte production [6]. Some data suggest the increased granulocyte mass typical of CML results from a few extra cell divisions within the hierarchy of granulopoiesis [5]. Untreated and/or absent effective therapy, *chronic phase* CML eventuates in uncontrolled proliferation, loss of differentiation and loss of response to normal control mechanisms. This phase of CML is termed *acute phase* or *blast crisis* and typically resembles acute myeloid leukemia (AML) or, less often, acute lymphoid leukemia (ALL). *Acute phase* is thought to result from additional genetic instability and acquisition of more mutations somehow caused by the activity of P210^*BCRABL1*^ [7].

Some persons appear to have a transition phase between *chronic* and *acute phases* termed as *accelerated phase*. Because of these diverse, arbitrarily defined, but not biologically based definitions of the *accelerated phase*, we consider CML a fundamentally biphasic disease. This is not a new concept. Many of the original CML chemotherapy trials done by CALGB divided the disease into *chronic* and *non-chronic phases*. There are many definitions of *accelerated phase* all of which are arbitrary, for example, defining *accelerated phase* by >10% blood or bone marrow blasts, >15% and >20% blood basophils, platelets <100 × 10E+9/L etc. means the same person can be in *chronic phas*e in one study and *accelerated phase* in another. Then, there is the obvious problem of someone saying that 19% blasts are in the *chronic phase* and the rest of them with 20% in the *accelerated phase*. There is no biological basis for such an arbitrary boundary. Adding to this, the daily variability of blast percent in someone with CML who could be in *chronic phase* one day, *accelerated phase* the next and back to *chronic phase* the next. And there is the problem of precision. Surveying 100–200 blood cells to determine percent blasts has reasonably wide confidence intervals, which can easily span any arbitrary boundary like 20%. Then we add to this inter-observer and intra-observer variability. The same arbitrariness applies to using additional cytogenetic abnormalities to define *accelerated phase*. Sandberg et al. reported that they could detect cytogenetic abnormalities used by some to define *accelerated phase* in many newly-diagnosed persons with CML when they surveyed 100 s of metaphases [8]. These persons typically had clinical features of *chronic* *phase* and most remained in chronic phase for years, sometimes decades. This is not surprising given the long latency from the start of CML to its diagnosis (see below). Others reported some, but not all additional cytogenetic abnormalities used to define *accelerated phase* are not associated with an increased risk of dying from CML [9, 10]. Hehlmann and co-workers recently reported some additional chromosome abnormalities used to define *accelerated phase* do not correlate with an increased probability of death survival in persons with CML [11]. The sum of these considerations supports the concept of CML as a bi-phasic disease.

The cell in which *BCRABL1* first occurs and which causes *chronic phase* CML is termed the CML leukemia stem cell (LSC). Some progeny of this cell may also have or acquire *stem cell* features including the biological ability to cause CML recurrence. As such there may eventually be more than 1 CML LSC in someone with CML, especially for a prolonged interval. However, there are several problems with this concept. First, there is substantial controversy over what feature(s) defines a *stem cell*. The most common definition is a cell which can continuously produce unaltered daughters, as well as daughter cells with different, more restricted properties. This contrasts with a *progenitor cell*, cells with proliferative capacity, which may or may not be committed to a lineage choice but are not terminally-differentiated. Adding to this complexity is the concept of precursor cells, cells which are usually, *although not always*, post-mitotic but have the capacity to assume one of several differentiated states. Whether CML starts in a *stem*, *progenitor*, or *precursor* cell is unknown and probably unknowable and unproveable. Although some may argue the presence of the Ph^1^-chromsome in other lineages such as B-cells proves CML must begin in a *stem cell*, we are not convinced considering the possibility *BCRABL1* may confer on a more mature *stem cell* features via de-differentiation much like occurs with induced pluri-potent stem cells [12]

A second cause of controversy is the definition of a *stem cell* varies based on the field of study, the organism being studied, the assay and other considerations. For example, the phenotype of a *stem cell* may not be static but vary at different points in the cell-cycle [13]. The same changeability may apply to definitions based on gene-expression profiling. A third consideration is that a cell which is not initially a *stem cell* may become a *stem cell* because of mutational, environmental, and/or architectural events (the bone marrow microenvironment).

Tyrosine kinase-inhibitors (TKIs) which block the biochemical activity of P210^*BCRABL1*^ can *cure* persons with *chronic phase* CML [14]. Whether this cure is *absolute*, namely no residual leukemia cells are able to cause CML recurrence even in someone with an infinite lifespan (sometimes equated, without convincing data, as no residual CML LSCs) or *operational* (no recurrence of CML during a person’s remaining lifetime) is controversial and we cannot distinguish these cures with present technologies. Another controversial issue is whether *operational* cure implies no recurrence of CML whilst receiving or not receiving therapy. For example, is someone without recurrent CML whilst on TKI-therapy cured? Probably no. We would not consider someone with diabetes cured just because their disease is controlled by taking insulin. Regardless of these complexities, life-expectancy of persons with *chronic phase* CML successfully-treated with TKIs is like sex-matched and age-matched normals in some, but not all studies [15, 16]. Most deaths in successfully-treated persons with CML are from the causes other than leukemia such as cardio-vascular disease and new cancers. Considerable conceptual and experimental data suggest that although TKIs inhibit proliferation of the CML clone they do not target CML LSCs [17].

About one-half of persons with CML achieving a deep molecular remission for a few years [about one-third to one-half of the whole population] can discontinue TKI-therapy without leukemia recurrence for median observation intervals up to 7–8 years [18, 19], a condition termed *therapy-free remission* (TFR). How this occurs is controversial. In some persons in TFR a low-concentration *BCRABL1* transcripts can be detected [20]. Whether this is important or convincing is difficult to know as there are several reports of detecting *BCRABL*1 transcripts in normals, presumably derived from cells without the biological capacity to cause CML [21, 22]. Another possibility is these transcripts are from the cells able to cause CML but do not do so within the observation interval, or perhaps within a person’s remaining lifetime. Stochastic considerations may also apply. Some studies report CML LSCs are present at diagnosis, during therapy and in persons who are in TFR. Other studies discussed the correlation between CML LSC detection and probability of achieving TFR, but this is controversial [23, 24].

Here, we review techniques to identify and quantify CML LSCs. We discuss possible mechanisms of resistance to TKIs and the potential influence of the bone marrow microenvironment on CML LSCs. These data may help develop strategies to target CML LSCs and perhaps cure more persons with CML.

## Cell surface antigens

A major challenge in studying LSCs is identifying a possible unique cell surface antigen phenotype. CML LSCs are CD34-positive and CD38-negative but this phenotype is not exclusive to CML LSCs [25]. Consequently, a useful cell surface antigen target must be on CML LSCs, but not normal stem cells or more differentiated leukemia cells, or must have a different expression pattern, density, or distribution. It must also be stably expressed on the CML LSC surface; antigens whose expression might vary with the cell-cycle would be less or not useful. An example is Siglec-3 (CD33) purportedly on normal stem cells and CML LSCs but with greater antigen density on CML LSCs [26]. Landberg et al. reported a similar disparity for CD36 [27]. Discriminating *normal stem cells* from CML LSCs may be difficult. Many other antigens such as CD44, CD47, CD52 are reportedly present on CML LSCs but also on normal hematopoietic stem/progenitor cells [28]. Other, supposedly, differentially expressed antigens include CD25, CD26, and interleukin-1-receptor accessory protein (IL-1RAP) [23, 29–31]. CD25 (IL2Rα) is regulated by STAT5 activity and increased CD25 expression is reported to reduce proliferation capacity of CML LSCs [32]. Also binding to IL-1RAP, a co-receptor of IL-1, activates the NF-kβ and AKT signaling pathways which increase proliferation of CML LSCs [33]. Some data suggest CD25 and IL-1RAP expression are unique to CML LSCs in CD34-postive, CD38-negative population but not in the more mature CD34-positive and CD38-positive fraction [29–31, 34].

Dipeptidyl peptidase-4, (DPP4; CD26) which cleaves diverse substrates such as chemokines and inhibits the stromal cell-derived factor-1 (SDF1; CXCL12). Cleaving the SDF1-CXCR4 axis by CD26 is implicated in releasing CML LSCs from the bone marrow into the blood and may be a marker of *chronic phase* CML LSCs [29, 35]. The concentration of CD26-positive CML LSCs in blood and bone marrow reportedly correlates with the white blood cells (WBC), but a relationship with response to TKI-therapy is unproved [29, 36]. CD26-positive LSCs are decreased after TKI treatment but following relapse or TKI-resistance number of CD26 expressing cells increases in the blood and bone marrow [29]. Similarly, Warfvinge et al. reported the concentration of CD26-postive LSCs correlates with TKI-resistance and identifies TKI-resistant sub-clones [37]. These authors also claim they can identify distinct sub-populations of CML LSCs based on single-cell gene expression patterns with CD26 expression restricted to resistant sub-clones [37]. The data we cite are complex and controversial. One possible profile of CML LSCs detected by flow cytometry could be cells which are Lin-negative, CD34-positive, CD38-negative/low, CD45RA-negative, *KIT*-negative, and CD26-positive [23, 37]. However, because we lack agreement on which biological assay defines a CML LSCs, it is presently impossible to know if this phenotype is correct. Newer studies suggest combining transcriptomics and proteomics data may be an effective approach in identifying antigens—qualitatively or quantitatively expressed on CML LSCs [38]. These data are displayed in Table 1.

**Table 1:** Differential expression pattern of surface markers on normal stem cells and CML LSCs

Marker	CD	CD34^+^/CD38^−^CML	CD34^+^/CD38^−^Normal	CD34^+^/CD38^+^CML	CD34^+^/CD38^+^Normal	Reference
IL-2Rα	CD25	++	–	+/−	+/−	[31]
DPP4	CD26	++	–	+/−	–	[29]
Siglec-3	CD33	++	+	+	++	[26]
SCARB3	CD36	++	+/−	++	++	[27]
IL-1RAP	–	+	–	+	+	[30]

## Bone marrow microenvironment

The bone marrow microenvironment is comprised of many cell-types including mesenchymal stromal cells, osteoblasts, osteoclasts, endothelial cells, and neural cells interact with normal haematopoietic stem cells through different molecules and signaling pathways [39–41]. Some data suggest levels of CXCR4, a chemokine receptor, correlate negatively with *BCRABL1* transcription and translation [42]. Several studies report inhibiting the tyrosine kinase activity of P210^*BCRABL1*^ reduces CXCR4 protein levels which might favor release of CML LSCs from the bone marrow *microenvironment* into the blood. Why inhibiting P210^*BCRABL1*^ decreases CXCR4 levels is unknown. In contrast, increased levels of CXCR4 protein triggers homing of CML LSCs to the bone marrow *microenvironment* which induces quiescence and TKI-resistance [42, 43]. Other selectin-related ligands like CD44 are impliacted in homing of CML LSCs. This activity is different from normal stem cells which depend on β1-integrins like Very Late Antigens-4 (VLA4), Very Late Antigens-5 (VLA5) [44, 45], and are home to the bone marrow microenvironment. Secretion of granulocyte-colony stimulating factor (G-CSF), an antagonist of SDF1, by CML LSCs may facilitate their release into the blood [46]. Similarly, increased expression of CD26 on CML LSCs interrupts the SDF1-CXCR4 interaction which may also release CML LSCs into the blood [29].

**Fig. 1:**
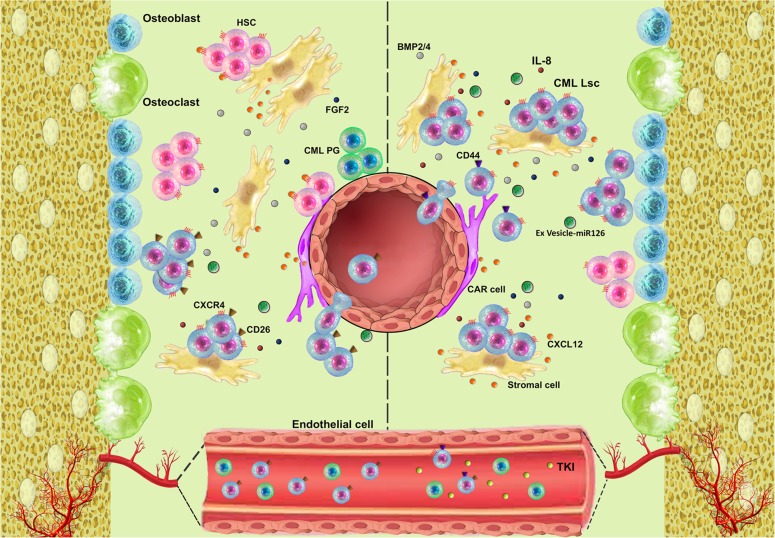
Structural and functional features of the bone marrow microenvironment. Before TKI-therapy downregulation of CXCR4 by P210^*BCRABL1*^ and increased expression of CD26 on CML LSCs causes them to exit the bone marrow and enter the blood. TKI-therapy reverses these effects causing CML LSCs are home to the bone marrow promoting their persistence

Cross-talk between CML LSCs and the bone marrow *microenvironment* is mediated by diverse molecules via paracrine and autocrine mechanisms. Some data suggest CML LSCs secrete exosomes containing amphiregulin, which activates the epidermal growth factor (EGFR) pathway in mesenchymal stromal cells [47]. This interaction increases secretion of IL-8 facilitating adhesion of CML LSCs to mesenchymal stromal cells and favouring their survival [47]. Other data suggest several mechanisms combine to promote resistance of CML LSCs to TKI-therapy. For example, increased expression of BMPR1b in CML LSCs cells and activation via BMP2/4 by autocrine and paracrine mechanisms involving mesenchymal stromal cells increases the expression of TWIST1 facilitating resistance to TKIs [48–50]. Other studies report a mesenchymal stromal cell-mediated decrease of reactive oxygen species (ROS) concentrations in CML LSCs or production of FGF2 by mesenchymal stromal cell increases TKI-resistance of CML LSCs [51, 52]. These concepts are displayed in Fig. 1.

## Effects of signaling pathways on CML LSCs

Mechanisms of resistance to TKIs include *BCRABL1*-dependent and *BCRABL1*-independent mechanisms [53–56] including mutations in the ATP-binding site or adjacent sites regulating the conformation of the dimeric P210^*BCRABL1*^ [57, 58] and the WNT/βcatenin, Hedgehog, PI3K/AKT and JAK/STAT signaling pathways.

β-catenin is important for self-renewal and long-term maintenance of normal *stem cells* and CML LSCs. Serial transplants of CML LSCs from β-catenin *null* mice into secondary recipients results in defects in self-renewal potential [59]. Also increased β-catenin expression is reported to correlate with advanced CML [60, 61]. Moreover, P210^*BCRABL1*^ interacts with β-catenin mediating its nuclear transition by stabilizing it [62]. One theory of TKI-resistance of CML LSCs is TKI-therapy amplifies CD70 expression by inhibiting miR29 which triggers WNT-signaling by activating CD27 [63]. Furthermore, interaction of mesenchymal stromal cells with CML LSCs via WNT/β-catenin signaling might cause TKI-resistance and increase proliferation of CML LSCs [64, 65]. Imatinib therapy increases activation of Nuclear Factor of Activated T-cells (NFAT), a transcription factor, by the non-canonical WNT signaling pathway resulting in increase pro-survival cytokines promoting imatinib resistance [66]. If so, therapy with a WNT/β-catenin-inhibitor and a TKI could eliminate CML LSCs [67, 68]. This remains to be proved.

Activation of Smoothened (Smo) by Ptch in the Hedgehog signaling pathway also activates Gli family transcription factors which stimulate transcription of target genes such as *GLI1*, *PTCH1*, *BCL2*, *CYCLIN D*, and *MYC* [69]. *Chronic phase* CML cells have high levels of mRNAs transcribed from genes in the Hedgehog signaling pathway suggesting a possible role in leukemia development [70]. Hedgehog signaling is activated in CML LSCs by upregulation of *Smo* persisting despite TKI-therapy suggesting this pathway is *BCRABL1*-independent [71]. Inhibiting Smo has no effect on normal HSCs but inhibits CML LSCs [71, 72]. One study reported exposing CML LSCs to cyclopamine, a Smo inhibitor, reduced their numbers and inhibited growth [73]. Another study reported PF-04449913, a Smo antagonist, causes cycling of CML LSCs and sensitizes them to TKIs [72].

The PI3K signaling pathway is important in maintaining normal *stem cells* and CML LSCs. One study reported PI3K signaling upregulates in CML LSCs [74] and correlates with P210^*BCRABL1*^ levels [75]. Normally, AKT phosphorylates FOXO transcription factors where it is sequestered in the cytoplasm. TKI-therapy promotes FOXO nucleus re-localization and restores transcriptional activity. Levels of BCL6, ATM, and CDKN1C, believed to be important for survival of CML LSCs, are increased by FOXO expression [76]. Another study reported inhibition of mTORC1 has no effect on CML LSCs whereas inhibiting PI3K increases susceptibility of CML LSCs to TKI-mediated inhibition [77]. Other data suggest arachidonate-15 lipoxygenase (Alox15) is essential for the maintenance of LSCs in a mouse model of CML. Inhibiting Alox15 expression increases PTEN expression, a negative regulator of PI3K-AKT, and downregulates expression of β-catenin, PI3K, and AKT [78].

**Fig. 2:**
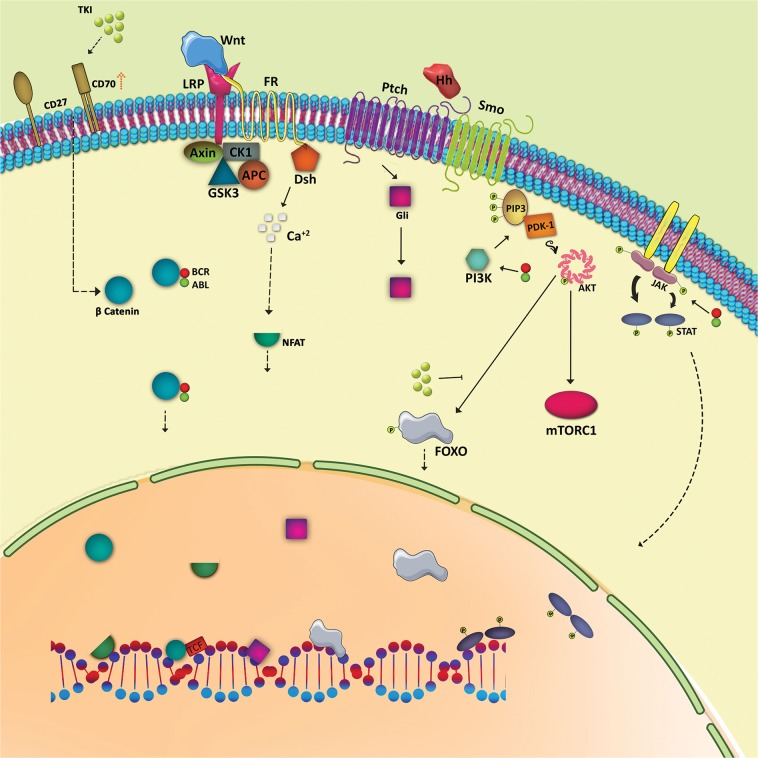
Possible signaling pathways in CML LSCs. Binding of WNT to the frizzled receptor and LRP as co-receptor and activation of WNT/β-catenin is considered the normal signaling mechanism. However, stabilization and transduction of β-catenin into nucleus by P210^*BCRABL1*^ and stimulation of CD70-CD27 after TKI-therapy may be specific to CML LSCs. Activation of WNT/Ca^2+^/NFAT favors CML LSC resistance to imatinib. Attachment of Hedgehog signaling ligands to the Ptch receptor activates Gli family transcription factors. Induction of PI3K activity by P210^*BCRABL1*^ phosphorylates PIP2 resulting in recruitment of PDK1 which phosphorylates AKT activating mTORC1 and sequesters FOXO transcription factors. TKI-therapy enhances nucleus localization of FOXOs increasing survival of CML LSCs. Activation of JAK/STAT signaling by P210^*BCRABL1*^ also increases survival of CML LSCs

The JAK/STAT signaling pathway may also be important in CML [79]. *BCRABL1* activates STAT1, STAT3, and STAT5 [80, 81]. Downstream oncogenic signaling and *JAK2* knockdown reduce P210^*BCRABL1*^ levels in *BCRABL1-*transfected mouse myeloid cells expressing *BCRABL1*-positive cell lines, including BV173, KBM-7, and K562-R [82]. *JAK2*-inhibition causes apoptosis of imatinib resistant CD34-positive CML cells from persons in *chronic and acute phases* [82]. Combining imatinib and interferon-γ decreases STAT5 phosphorylation whilst increasing phosphorylation of STAT1. This increases LSC survival, likely by upregulating expression of *BCL6* [83]. Ruxolitinib, a *JAK2*-inhibitor, with nilotinib may decrease CML LSCs whilst sparing normal *stem* cells [84]. This remains to be proved. Signaling pathways in CML LSC are displayed in Fig. 2.

## microRNAs and CML LSCs

Some data suggest microRNAs may be important in CML LSC self-renewal, maintenance, and perhaps TKI-resistance [85]. miR-126 is reported to regulate dormancy of normal stem cells and CML LSCs [86]. P210^*BCRABL1*^ phosphorylates SPRED1 which downregulates miR-126 resulting a loss of *stemness*. In contrast, bone marrow endosteal Sca-1 + endothelial cells secrete high amounts of miR-126 via extra-cellular vesicles. TKIs reverse miR-126 inhibition with miR-126 levels increased by TKI-therapy. Decreased miR-126 levels sensitize CML LSCs to TKI [86]. Increased JAK-STAT signaling mediated by P210^*BCRABL1*^ (discussed above) increases ADAR1 levels increasing metabolism of adenosine to inosine which regulates miRNA stability. ADAR1 impairs biogenesis of mir-let7, a miR precursor, increases self-renewal of CML LSCs [87]. Exposing CML LSCs to imatinib increases levels of miR-21 resulting in TKI-resistance [88]. Interruption of the PI3K/AKT signaling pathway depletes miR-21 by amplifying PDCD4 and PTEN restoring sensitivity of CML LSCs to TKIs [88]. Proliferation of CML LSCs by E2F1 is mediated by increased levels of miR183 [89]. Also, miR-30a levels are decreased after imatinib-therapy by a mechanism involving Beclin1 and ATG5, which favors LSC resistance to TKIs [90]. Other miRNAs unrelated to P210^*BCRABL1*^ may also operate [91]. Increases in miRs-29a and miRs-29a-660 which target *TET2* and EPAS1 decrease miR-494 thereby increasing CML LSC TKI-resistance [91]. Whether long noncoding RNAs (lncRNAs) and circular RNAs (circRNAs) are important in CML LSC biology remains unstudied. These data are displayed in Table 2.

**Table 2:** Expression pattern and role of relevant miroRNAs in CML LSCs

MicroRNAs	Role	Expression	BCR-ABL dependency	Reference
MiR-126	Dormancy	↑	+	[86]
MiR-let7	Tumor suppressor	↓	+	[87]
MiR-21	Drug resistance	↑	+	[88]
MiR-183	Proliferation	↑	+	[89]
MiR-30a	Drug resistance	↑	+	[90]
MiR-29a	Drug resistance	↑	–	[91]
MiR-660	Drug resistance	↑	–	[91]
MiR-494	Tumor suppressor	↓	–	[91]

## Autophagy and CML LSCs

**Fig. 3:**
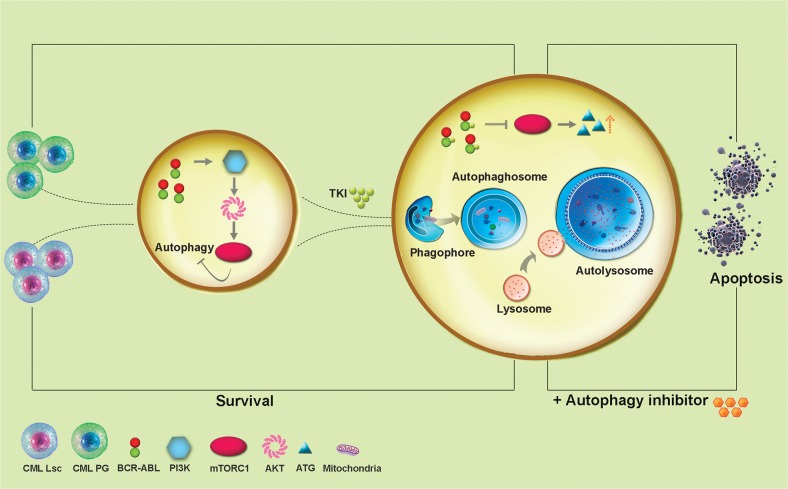
Autophagy in CML. The interaction of P210^*BCRABL1*^ with PI3K-AKT inhibits autophagy. Contrariwise, TKIs inhibit kinase activity of P210^*BCRABL1*^ enhancing autophagy. These autophagy effects may kill CML progenitor cells but may preserve CML LSCs

Several studies report complex, contradictory effects of TKI-therapy on autophagy in CML. For example, imatinib therapy increases intra-cellular levels of ATG4B which sensitizes CML LSCs cells to TKIs [92]. P210^*BCRABL1*^ increases levels of ATF5 via the PI3K/AKT pathway which upregulates mTORC1, an autophagy inhibitor. In contrast, inhibition of kinase activity of P210^*BCRABL1*^ by TKIs downregulates the PI3K/AKT pathway thereby increasing autophagy of CML progenitor cells but not CML LSCs [93–96]. Whether survival of CML LSCs can be overcome by adding other drugs is unknown [97]. In total, the role of autophagy in the biology of CML LSCs is complex and poorly understood. These concepts are displayed in Fig. 3.

## Other molecules potentially involved in survival of CML LSCs

### Blk

Concentrations of Blk, a tyrosine-kinase, are lower in CML LSCs compared with normal stem cells. Blk is reported to suppress CML LSCs by upregulating p27. However, P210^*BCRABL1*^ decreases Blk expression by modulating Pax5. Over-expression of Blk in CML LSCs inhibits self-renewal and increases apoptosis whereas Blk knock-down has no effect on normal *stem cells* [98].

### *EZH2*

*EZH2*, part of the PRC2 complex, is an epigenetic repressor operating by tri-methylation of histone H3 (H3K27me3). *EZH2* is upregulated in CML LSCs and is downregulated by TKI-therapy. Some data suggest inhibiting *EZH2* increases the likelihood of eradicating CML LSCs with TKI-therapy whilst sparing normal *stem cells* [99] but this finding needs confirmation.

### Fap-1

Fap-1 is a phosphatase which inhibits Fas-mediated apoptosis and stabilizes β-catenin by targeting Gsk3β, a β-catenin inhibitor. Increased Fap-1 levels are associated with persistence of CML LSCs. Inhibiting Fap-1 by Fap-1 blocking tripeptide in mice with a bone marrow transduced with a cDNA to human *BCRABL1* increases response to TKIs and inhibits leukemia progression [100].

### HIFs

Levels of hypoxia inducible factors (HIFs) increase in hypoxia conditions. This increase inhibits normal stem cell differentiation promoting a quiescent state [101]. HIF-1, a transcription factor, is important in regulating proliferation, maintenance, and survival of CML LSCs. Cheloni et al. reported acriflavine, a HIF-1 inhibitor, targets CML LSCs by reducing *MYC* and decreases *stemness*-related genes such as NANOG, SOX2, and OCT4 by decreasing HIF-2α. CML LSCs are more dependent on HIF-1 than normal HSCs. Consequently, combining a HIF-1 inhibitor with a TKI could potentially target CML LSC resident in hypoxic regions in the bone marrow microenvironment [102, 103]. Whether this is so requires confirmation.

### PML

PML (promyelocyte leukemia protein) forms PML-nuclear bodies (PML-NBs) which are involved in multiple genome maintenance pathways including the DNA damage response and repair, telomere homeostasis, and p53-associated apoptosis. PML-NBs also play a role in repairing DNA double-strand breaks [DSBs] by homologous recombination [104]. Upregulation of PML in CML LSCs prevents cycling thereby increasing resistance to TKIs. Targeting PML in acute promyelocytic leukemia (APL) with all-trans retinoic acid and/or arsenic trioxide results in PML degradation and triggers cycling of the quiescent APL LSCs. This strategy could help eradicate CML LSCs by restoring TKI-sensitivity [105]. Interestingly, various forms of arsenic were used to treat persons with CML in the early 20th century. There are several ongoing and complete clinical trials of arsenic trioxide and TKIs in persons with CML (https://clinicaltrials.gov/ct2/results?cond=Chronic+Myeloid+Leukemia&term=arsenic&cntry=&state=&city=&dist=)

### PP2A

Protein phosphatase 2A (PP2A), a serine/threonine phosphatase, is involved in the β-catenin pathway, programmed cell death, and cell cycle progression [106]. Decreasing PP2A in CML LSCs stimulates self-renewal [107]. The several isoforms of PP2A have diverse stimulatory and inhibitory effects on cancer cells [106]. Recently, Lai et al. reported combining a PP2A-inhibitor with a TKI suppresses CML [107, 108].

### ALOX5

*ALOX5* encodes 5-lipoxygenase (5-LO) which converts arachidonic acid into leukotrienes and is involved in inflammation and cancer development [109]. *ALOX5* is important for induction of CML in mice [110]. Inhibiting ALOX5 with zileuton selectively reduces survival of CML LSCs compared with normal mouse stem cells [110]. In contrast to mice, humans have low *ALOX5* expression and zileuton is inactive [111]. These data suggest targeting ALOX5 is probably not an effective strategy in humans.

### SIRT1

Sirtuin 1 (SIRT1), a histone deacetylase, regulates gene expression, metabolic activity, and aging [112]. SIRT1 over-expression in CML LSCs deacetylates many transcription factors including P53, Ku70, and FOXO1 promoting drug resistance and survival of CML LSCs [113, 114]. Targeting SIRT1 in CML LSCs enhances acetylation of P53 increasing apoptosis [115]. This approach is untested in humans.

### BCL2

Levels of *BCL2*-related anti-apoptotic proteins are reported to be expressed at higher levels in CML LSCs compared with normal stem cells [116] and is further increased in *acute phase*. Some data suggest P210^*BCRABL1*^ activates downstream signaling pathways such as PI3K and JAK/STAT resulting in TKI-resistance [117]. Some data suggest inhibiting *BCL2* using subutoclax increases killing of CML LSCs by TKIs [116]. Another study in transgenic mice reported *acute phase* CML LSCs express high levels of BCL-xl, MCL-1, and BCL2. Venetoclax, another *BCL2*-inhibitor, combined with a TKI reduces the serial re-engraftment capacity of CML LSCs in mice compared with a TKI alone [118]. Clinical trials of venetoclax and a TKI in *chronic* (NCT02689440) and *acute phase* CML (NCT03576547) are beginning.

**Fig. 4:**
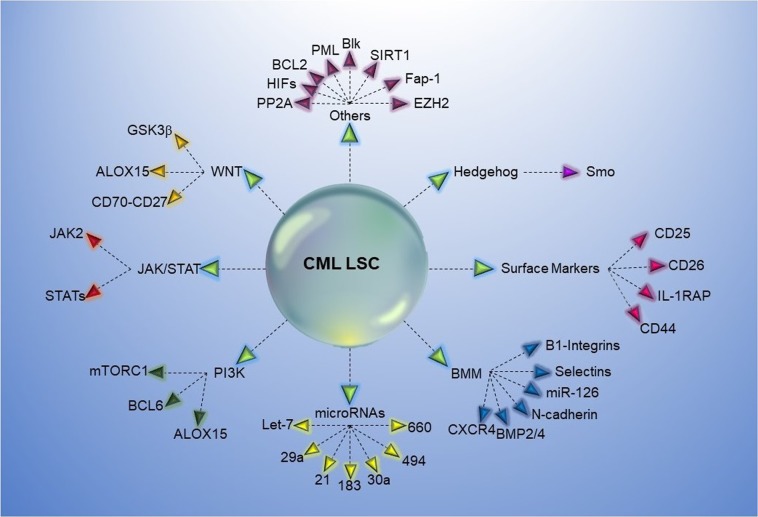
Potential molecules and pathways to target CML LSCs

All potential molecules for targeting CML LSC are displayed in Fig. 4.

## Metabolomics of CML LSCs

Metabolic re-programming of CML LSCs in the hypoxic bone marrow microenvironment is a proposed mechanism of *BCRABL1*-independent TKI-resistance of CML LSCs. The concentration of intra-cellular dipeptide amino acids is increased in CML LSCs compared with normal stem cells. This effect is thought to be the results of upregulation of SLC15A2, a peptide transporter. Dipeptides activate the p38MAPK pathway resulting in phosphorylation of Smad3, important in maintaining CML LSCs [119].

Prostaglandin E1 (PGE1) an E2 (PGE2) are derived from di-homo-γ-linolenic acid [120]. PGE2 promotes β-catenin signaling in CML LSCs. In contrast, PGE1s via its interaction with E-type prostanoid receptor 4 (EP4) suppresses the self-renewal of CML LSCs and inhibits their engraftment in immune deficient mice [121].

CML LSCs have increased lipolysis and fatty acid oxidation with increased levels of glycerol-3-phosphate, carnitine, and acylcarnitine derivatives and decreased free fatty acids compared with differentiated CML cells. Consequently, inhibiting this pathway could be a therapy target [122, 123]. CML LSCs also have increased mitochondrial oxidative phosphorylation mediated by upregulation of genes involved in oxidative metabolism [122]. Single-cell transcriptome data suggest over-expression of oxidative phosphorylation and glycolysis-associated genes in CML LSCs compared with normal stem cells [124].

Under physiological hypoxic conditions (PO2 < 32 mmHg), HIF-1α signaling is crucial for the survival of CML LSCs treated with TKIs [125]. Short-term culture of CML LSCs under hypoxic conditions induces upregulation of genes involved in carbohydrate metabolism. These data suggest HIF-1α-driven glycolytic adaptation to support energy production [125].

In summary, there are some differences in the metabolomics of CML LSCs compared with normal stem cells. However, these pathways are poorly-defined and there are no data targeting the metabolism of CML LSCs alone or with TKIs is a reasonable therapy target.

## Is targeting CML LSCs clinically important?

Above we discuss detailed studies of CML LSCs including their conclusions and limitations. Despite this extraordinary interest in CML LSCs—the question arises: is any of this research clinically important and do we have a correct definition of what is a CML LSC?

Data from several clinical trials indicate it’s possible to stop TKI-therapy in about one-half of persons with CML achieving a deep molecular response for an interval without CML recurrence for an extended subsequent interval, a situation referred to as therapy-free remission (TFR) [19, 126, 127]. Exactly what duration of deep molecular response is associated with the highest probability of TFR which is controversial. It is unlikely any specific duration is *best*. More likely, different durations of DMR will be associated with different probabilities of achieving TFR. However, these data coming from cohort studies, are complicated by *guarantee time bias* and will have wide 95% confidence intervals, limiting utility as applied to individuals [128]. That longer deep molecular remission intervals are associated with higher probabilities of TFR is a self-fulfilling prophesy which remains to be proved in a randomized clinical trial.

Considerable data indicate most if not all persons in TFR have some or even many residual CML LSCs as currently-defined including detection of *BCRABL1* transcripts with sensitive technique such as digital polymerase chain reaction [23, 24]. These data support two conclusions: (1) it may not be necessary to eradicate all CML LSCs to achieve TFR in at least some people with CML; and (2) some or perhaps all of what we currently define as CML LSCs are resistant to killing by TKIs and can persist in persons in deep molecular remission including those able to stop TKI-therapy without CML recurrence. It is also possible that our definition[s] of a CML LSC is wrong and the cells we are measuring as CML LSCs are not.

What is the nature of the residual CML cells in persons in TFR? Are they really CML LSCs or CML progenitor cells with residual clonogenic activity or which have acquired *stemness* and revert to being LSCs? Why do some persons with deep molecular response who stop TKI therapy cause CML recurrence and in others not? Does everyone stopping TKI-therapy have residual CML LSCs? Could leukemia recurrence be a stochastic event or will everyone have leukemia recurrence if followed for enough interval? The latter theory is consistent with radiation-induced CML in the A-bomb survivors with median latencies for males and females of 13 and 17 years after exposure [16]. Or does the immune system keep residual CML LSCs in check as some have suggested? [129, 130]. Here, we must contend with contradictory data. There is a strong anti-CML-effect detected after allogeneic haematopoietic cell transplants [131]. Whether this anti-leukemia effect is distinct from graft-versus-host disease (G*v*HD) is controversial and little anti-leukemia effect occurs after transplants from genetically-identical twins [131]. It is also noteworthy that neither the CML is increased in persons with inherited, congenital, or acquired immune deficiency diseases nor in immune-suppressed recipients of solid organ transplant [132].

## Conclusion

Identifying and understanding the biology of CML LSCs is an interesting and important area for scientific research. There are considerable data in this regard including >1500 articles published since 2000. Some of these provide valuable insights into the biology of CML LSCs, some are of questionable value and others are contradictory and/or mis-leading. There are important biological insights in many of these typescripts but an over-riding question is what the clinical importance of this research? For example, some people claim the cure of CML with TKIs would not be possible without a knowledge of CML LSCs. No scientific data support for this claim. The success of TKI-therapy hinged on measuring *BCRABL1* transcript levels, not quantifying CML LSCs. These transcript are probably not transcribed by CML LSCs. Perhaps, if we could accurately and precisely identify and quantify CML LSCs, we might be able to cure more persons with CML. Time will tell if the investment in studying CML LSCs will be of clinical value.
